# Supplementary material for: Differential Gene Expression Correlates with Behavioural Polymorphism during Collective Behaviour in Cockroaches
Source: Animals (Basel). 2022 Sep 8;12(18):2354. doi: 10.3390/ani12182354 (PMC9495117; doi:10.3390/ani12182354)
Supplement: Supplementary file 1 [file animals-12-02354-s001.zip › FileS1_BUSCO_GENEWIZ.pdf]

BUSCO version is: 5.2.2

The lineage dataset is: **eukaryota_odb10** (Creation date: 2020-09-10, number of genomes: 70, number of BUSCOs: 255)

***** Results: *****
C:65.5%[S:65.5%,D:0.0%],F:17.6%,M:16.9%,n:255
167 Complete BUSCOs (C)
167 Complete and single-copy BUSCOs (S)
0 Complete and duplicated BUSCOs (D)
45 Fragmented BUSCOs (F)
43 Missing BUSCOs (M)
255 Total BUSCO groups searched

The lineage dataset is: **insecta_odb10** (Creation date: 2020-09-10, number of genomes: 75, number of BUSCOs: 1367)

***** Results: *****
C:72.4%[S:71.4%,D:1.0%],F:11.6%,M:16.0%,n:1367
990 Complete BUSCOs (C)
976 Complete and single-copy BUSCOs (S)
14 Complete and duplicated BUSCOs (D)
158 Fragmented BUSCOs (F)
219 Missing BUSCOs (M)
1367 Total BUSCO groups searched

# Total library size of our samples:

SRT 1	24342808
SRT 2	26949490
SRT 3	25365913
LRT 1	26131017
LRT 2	24808578
LRT 3	25309377

GENEWIZ, Inc.
115 Corporate Boulevard
South Plainfield, NJ 07080
p (877) GENEWIZ (436-3949)
f (908) 333-4511
www.genewiz.com

2. NGS Data Overview

2.1 Data Summary

Project	Sample	Barcode sequence	PF Clusters	Yield (Mbases)	% >= Q30 bases	Mean Quality Score
DW1611142-R1	1	CGATGT	44,472,350	8,983	94.57	35.74
DW1611142-R1	2	TGACCA	46,076,550	9,307	94.63	35.75
DW1611142-R1	3	ACAGTG	43,323,118	8,751	94.56	35.74
DW1611142-R1	4	GCCAAT	45,647,723	9,221	94.55	35.73
DW1611142-R1	5	CAGATC	42,119,460	8,508	94.68	35.76
DW1611142-R1	6	CTTGTA	42,835,634	8,653	94.64	35.75

2.2 Q score distribution

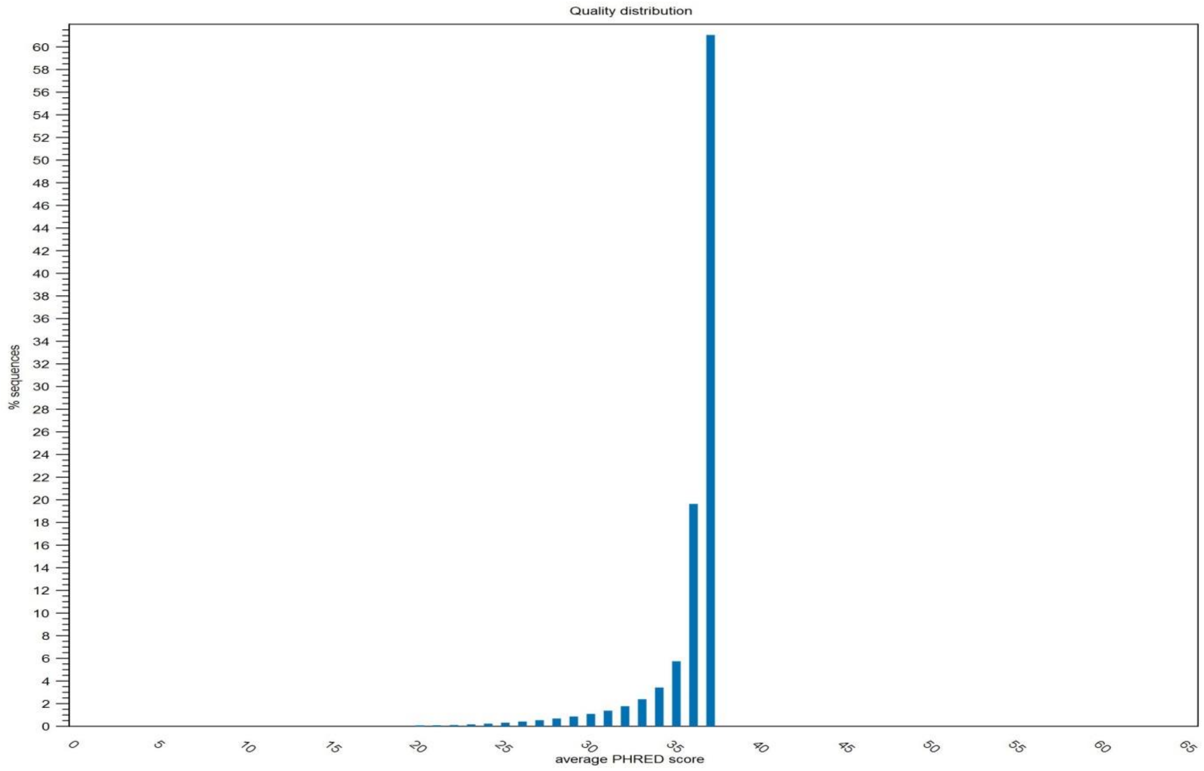

Figure 2.2 Q-Score distribution of all samples. X-axis, average Q scores. Y-axis, the percentage of sequence data.

GENEWIZ, Inc.
115 Corporate Boulevard
South Plainfield, NJ 07080
p (877) GENEWIZ (436-3949)
f (908) 333-4511
www.genewiz.com

3. Assembly Analysis

3.2 De novo transcriptome assembly

Sequence reads were trimmed to remove possible adapter sequences at the 3' ends and nucleotides with poor quality (error rate < 0.01) with CLC Genomics Server 8.0. As a result, 61430 transcript sequences were obtained. The mean size of assembled transcripts was 1258bp and the longest transcript was 18670bp. The total length of all transcripts was 77.3 Mbp. Consensus sequences for the assembled transcripts were obtained and blasted against NCBI nt database. In addition, Open-Reading-Frames were predicted from the assembled transcripts.

3.2.1 Nucleotide distribution in assembled sequences

Nucleotide	Count	Frequency
Adenine (A)	24,378,844	31.60%
Cytosine (C)	14,261,911	18.50%
Guanine (G)	14,285,016	18.50%
Thymine (T)	24,339,631	31.50%

3.2.2 Assembled transcript measurements

	Length
N75	885
N50	1,466
N25	2,574
Minimum	452
Maximum	18,670
Average	1,258
Count	61,430
Total	77,265,402
