# Supplementary material for: Differential Gene Expression Correlates with Behavioural Polymorphism during Collective Behaviour in Cockroaches
Source: Animals (Basel). 2022 Sep 8;12(18):2354. doi: 10.3390/ani12182354 (PMC9495117; doi:10.3390/ani12182354)
Supplement: Supplementary file 1 [file animals-12-02354-s001.zip › Supplementary_Figs_tables.pdf]

Differential gene expression correlates with behavioural polymorphism during collective behaviour in cockroaches

Isaac Planas-Sitjà*[1]; Jean-Louis Deneubourg[3]; Denis L. J. Lafontaine[2]; Ludivine Wacheul[2]; Adam L. Cronin[1]

*correspondence author: iplanass@pm.me

[1]Department of Biology, Tokyo Metropolitan University, 1-1 Minami-Osawa, Hachioji, Tokyo, 192-0397, Japan
[2]RNA Molecular Biology, Université libre de Bruxelles, Fonds de la Recherche Scientifique (F.R.S./FNRS), 6041 Gosselies, Belgium
[3]Center for Nonlinear Phenomena and Complex Systems (CENOLI) - CP 231, Université libre de Bruxelles, Campus La Plaine NO.5, Boulevard du Triomphe, 1050 Bruxelles, Belgium

Supplementary material

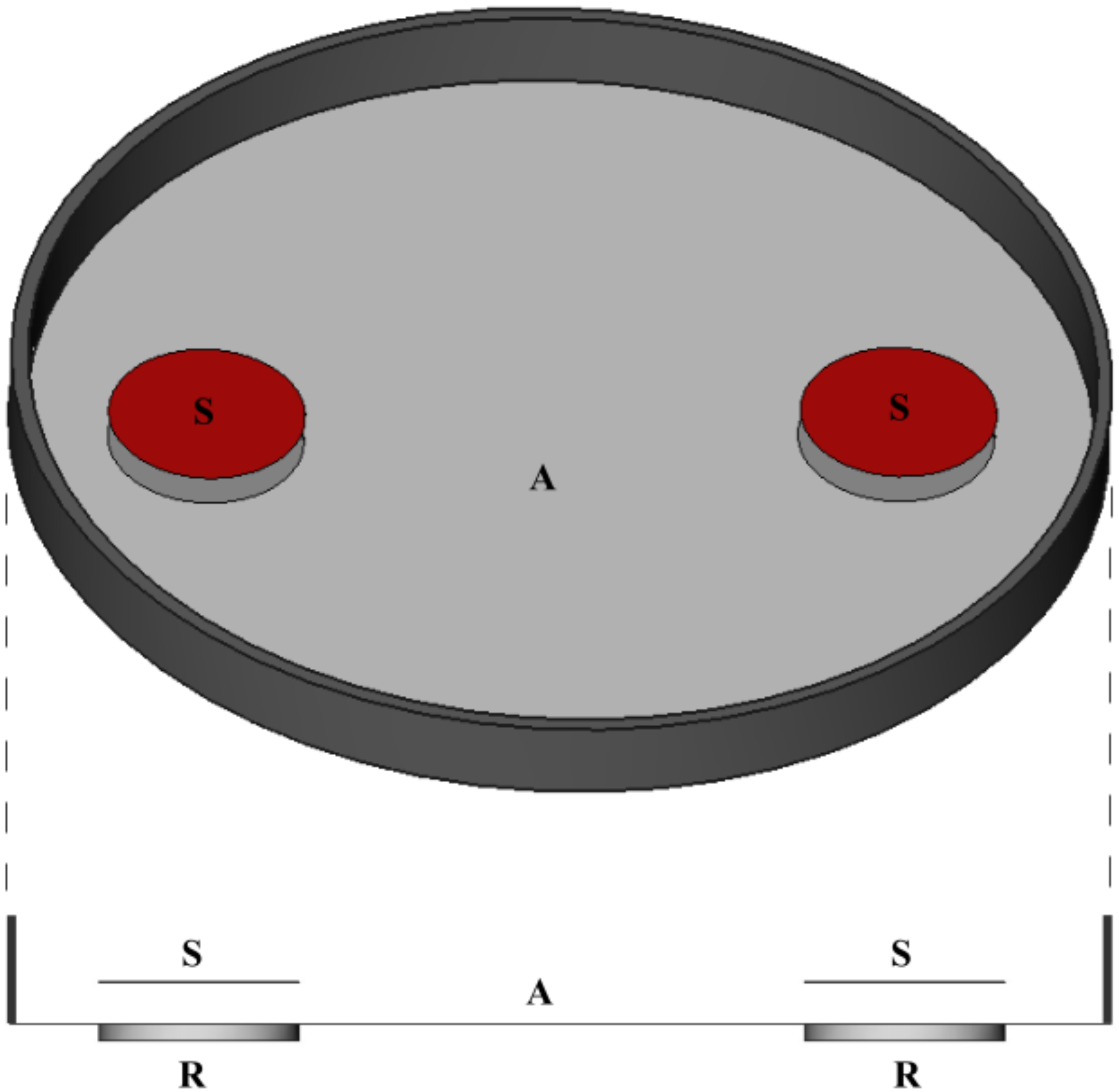

Fig. S1: Design of the experimental set-up in a perspective and latral view. A: Arena of the setup; S: Shelters with red filter; R: RFID reader.

Figure from Planas-Sitjà et al. (2015). Group personality during collective decision-making: a multilevel approach. Proc Roy Soc B, 282 (20142515). doi: 10.1098/rspb.2014.2515

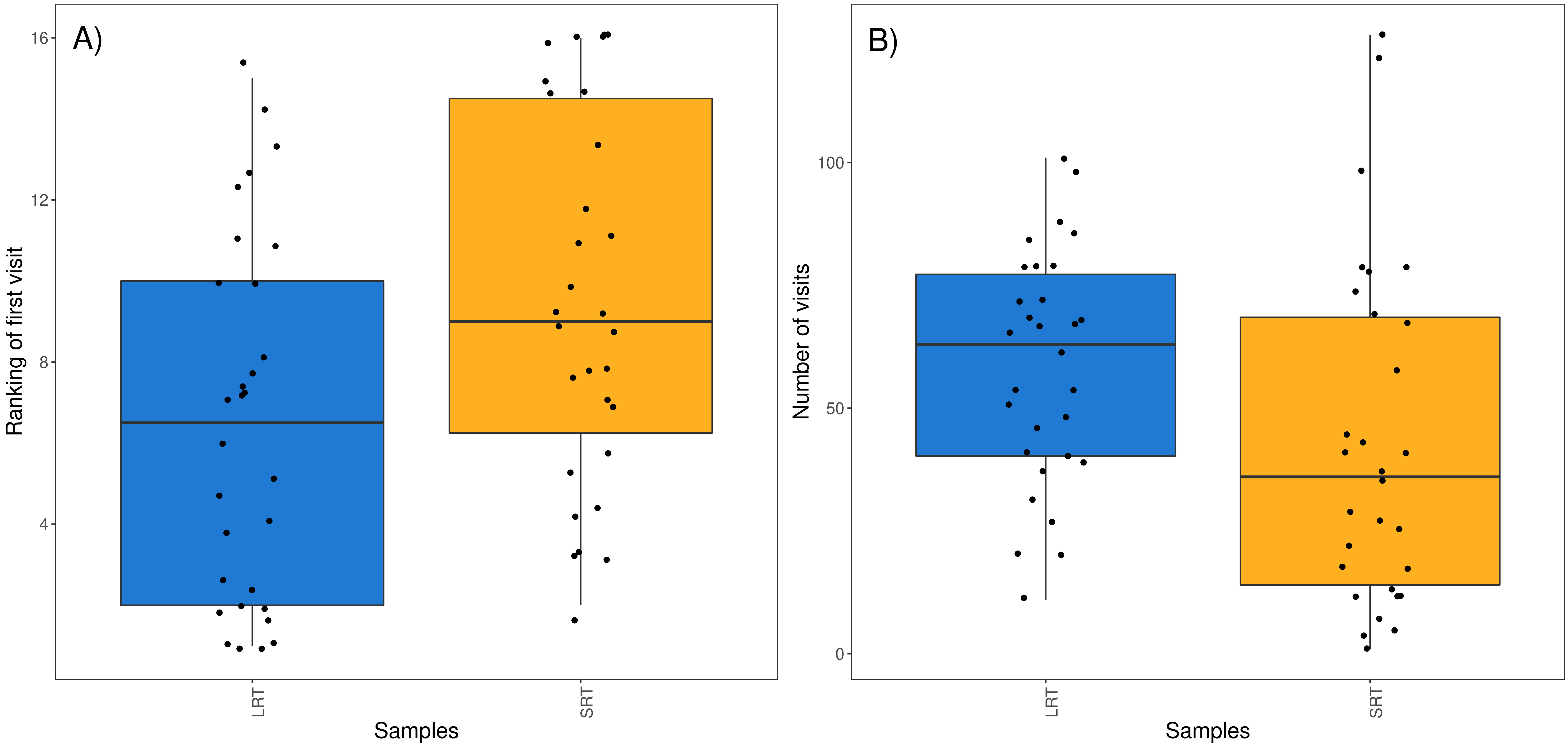

Fig. S2: A) Comparison of ranking of first visit (ie. order to visit the shelter for first time) between LRT and SRT individuals, and B) comparison of the number of visits to shelters between LRT and SRT individuals.

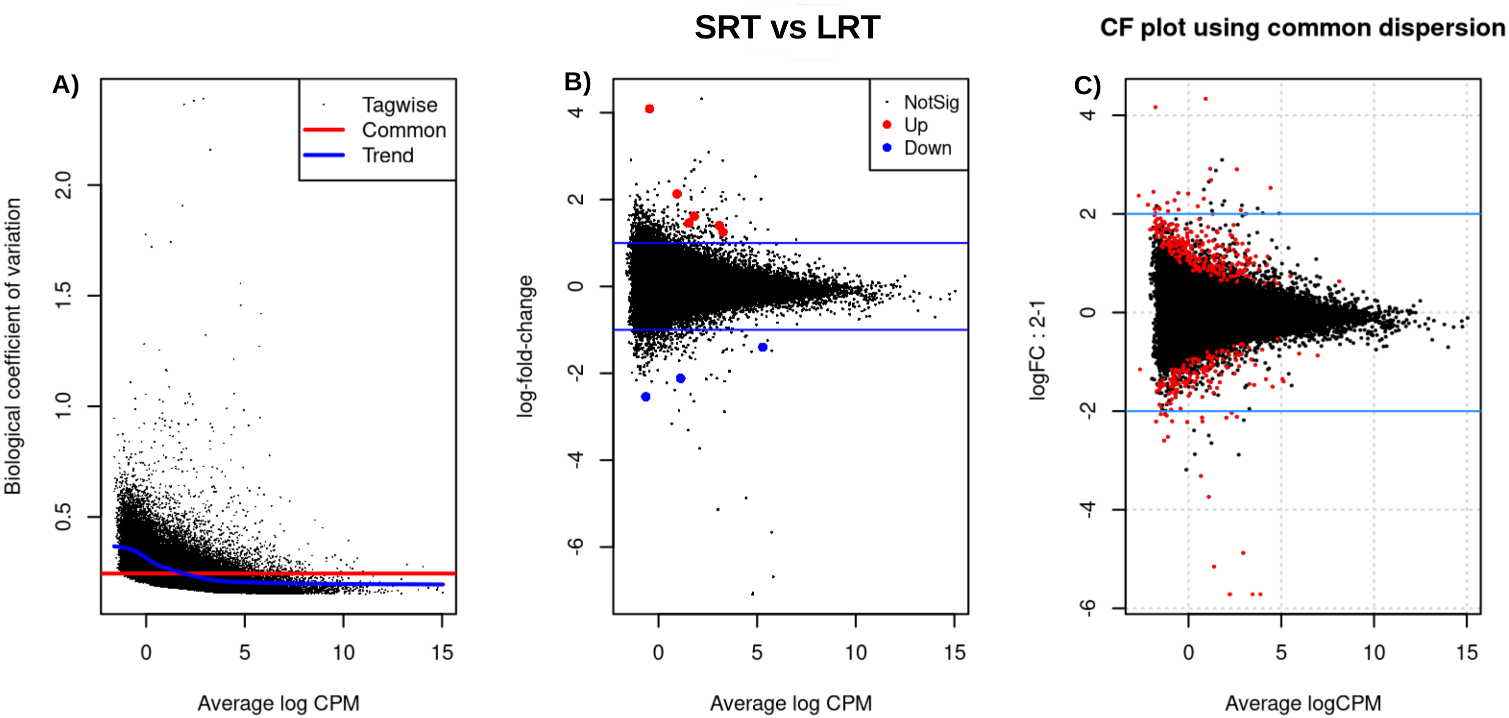

Fig. S3: A) Biological coefficient of variation for all transcripts. B) Significant transcripts after correction for multiple testing (see main text). We obtained 6 transcripts up-regulated in SRT (red) and 3 in LRT (blue). C) Significant transcripts with P < 0.01 (red).

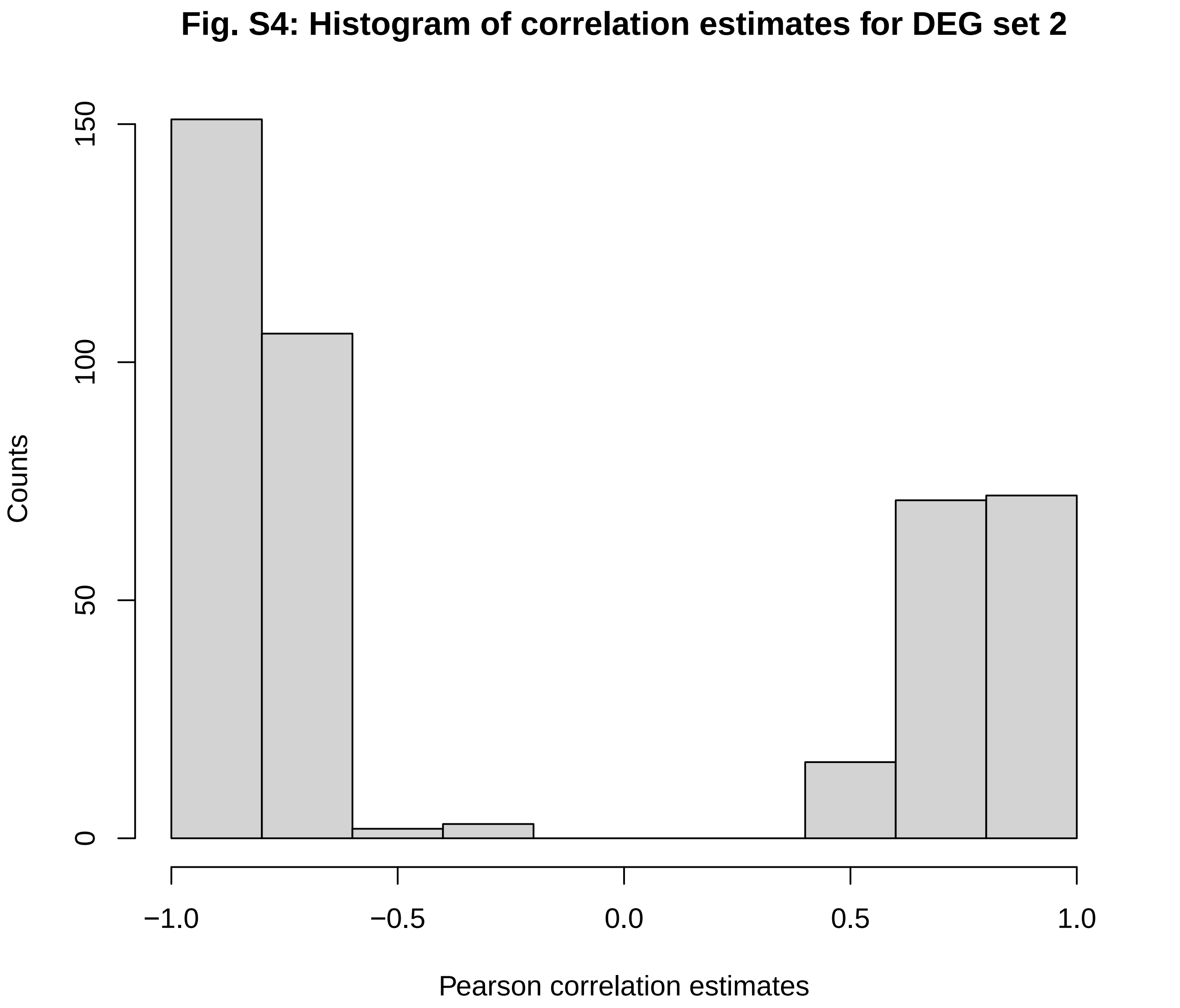

Fig. S4: Histogram of Pearson correlation estimates (r) between CPM of 421 transcripts in DEG set 2 and mean resting times of our six samples

Fig. S5: Subgraph with all significant genes

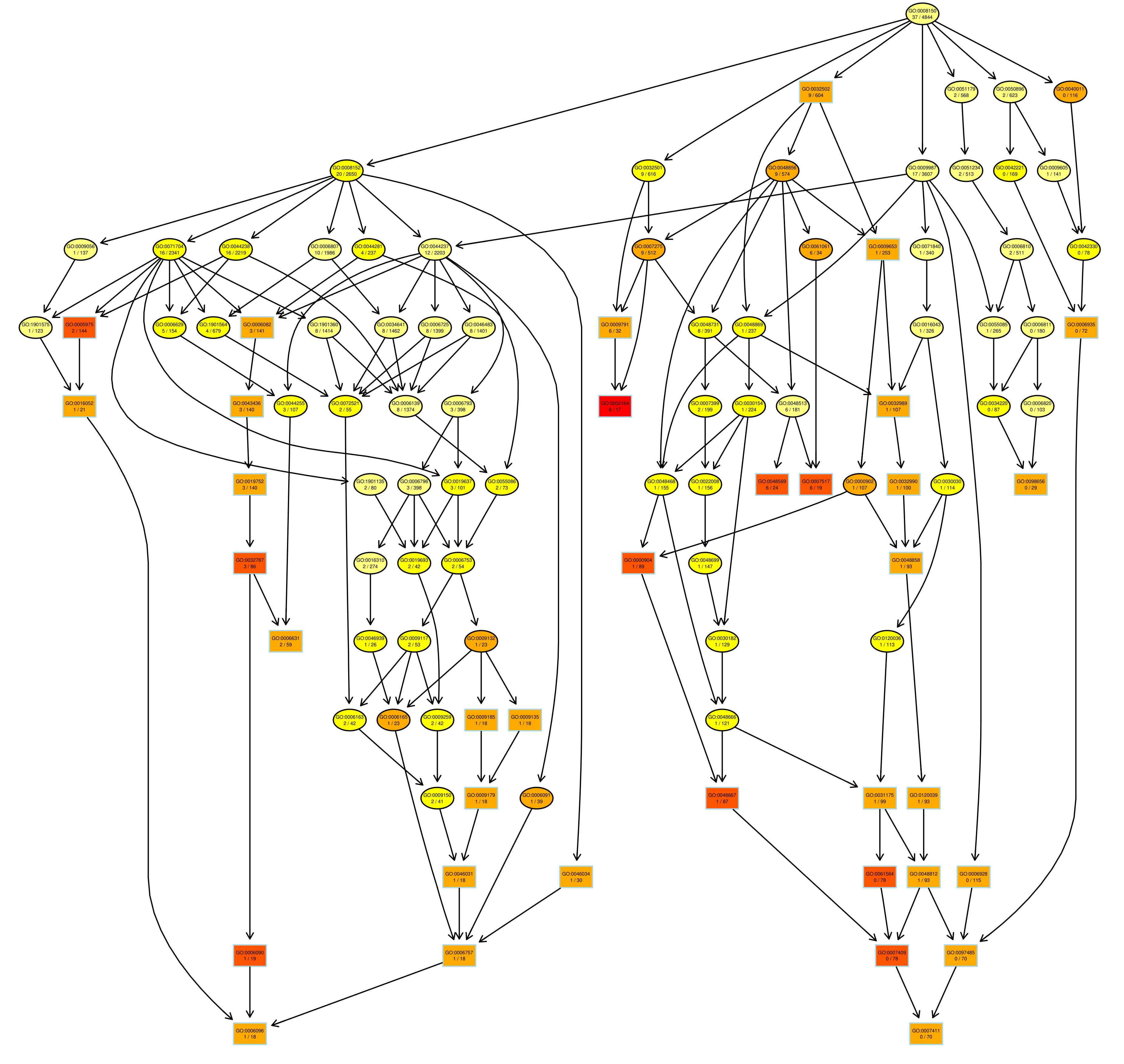

Fig. S6: Subgraph with top 10 significant genes

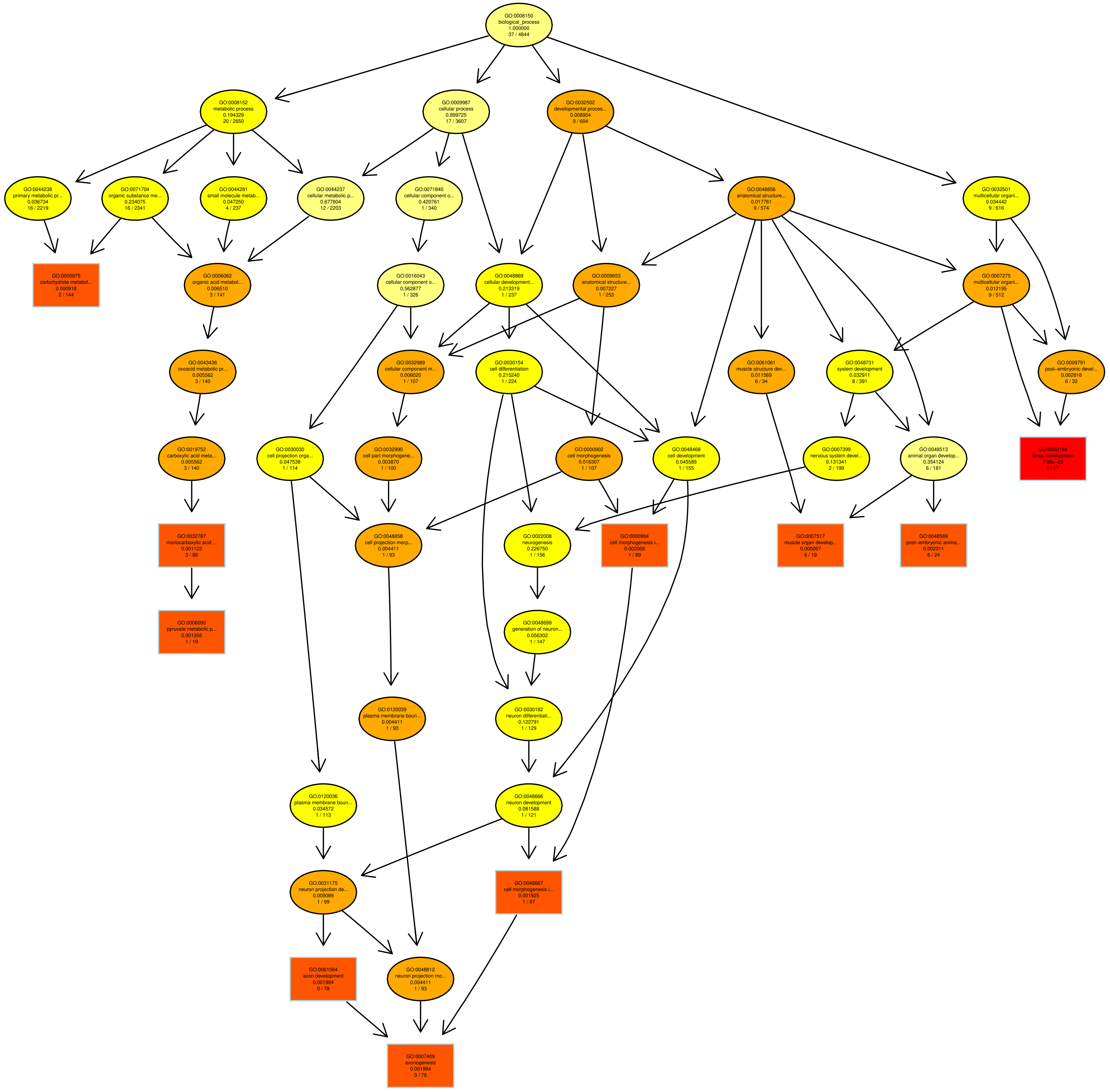

Table S1: Dunn comparison among SRT and LRT samples

	Comparison	Z	P.unadj	P.adj
1	LRT1 – LRT2	–0.56453449	0.5723904365	0.660450504
2	LRT1 – LRT3	–0.57910781	0.5625164317	0.703145540
3	LRT2 – LRT3	0.01319909	0.9894689541	0.989468954
4	LRT1 – SRT1	2.06178487	0.0392282222	0.073552917
5	LRT2 – SRT1	3.40129100	0.0006706839	0.003353420
6	LRT3 – SRT1	3.68782881	0.0002261757	0.003392636
7	LRT1 – SRT2	2.25671086	0.0240261479	0.051484603
8	LRT2 – SRT2	3.38092996	0.0007224096	0.002709036
9	LRT3 – SRT2	3.57861849	0.0003454153	0.002590614
10	SRT1 – SRT2	0.53099079	0.5954251611	0.637955530
11	LRT1 – SRT3	1.52147415	0.1281408933	0.213568155
12	LRT2 – SRT3	2.64641793	0.0081349242	0.020337310
13	LRT3 – SRT3	2.84420142	0.0044522893	0.013356868
14	SRT1 – SRT3	–0.68977727	0.4903342645	0.668637633
15	SRT2 – SRT3	–1.08408883	0.2783254146	0.417488122

Table S2: List of GO terms enriched

	GO.ID	Term	Annotated	Significant	Expected	classicKS
1	GO:0002164	larval development	17	6	0.13	7.9e−05
2	GO:0007517	muscle organ development	19	6	0.15	0.00027
3	GO:0005975	carbohydrate metabolic process	144	2	1.10	0.00092
4	GO:0032787	monocarboxylic acid metabolic process	86	3	0.66	0.00112
5	GO:0006090	pyruvate metabolic process	19	1	0.15	0.00136
6	GO:0048667	cell morphogenesis involved in neuron di...	87	1	0.66	0.00193
7	GO:0007409	axonogenesis	78	0	0.60	0.00199
8	GO:0061564	axon development	78	0	0.60	0.00199
9	GO:0000904	cell morphogenesis involved in different...	89	1	0.68	0.00207
10	GO:0048569	post−embryonic animal organ development	24	6	0.18	0.00231
11	GO:0009791	post−embryonic development	32	6	0.24	0.00282
12	GO:0007411	axon guidance	70	0	0.53	0.00316
13	GO:0097485	neuron projection guidance	70	0	0.53	0.00316
14	GO:0006096	glycolytic process	18	1	0.14	0.00322
15	GO:0006757	ATP generation from ADP	18	1	0.14	0.00322
16	GO:0009135	purine nucleoside diphosphate metabolic ...	18	1	0.14	0.00322
17	GO:0009179	purine ribonucleoside diphosphate metabo...	18	1	0.14	0.00322
18	GO:0009185	ribonucleoside diphosphate metabolic pro...	18	1	0.14	0.00322
19	GO:0046031	ADP metabolic process	18	1	0.14	0.00322
20	GO:0032990	cell part morphogenesis	100	1	0.76	0.00387
21	GO:0048812	neuron projection morphogenesis	93	1	0.71	0.00441
22	GO:0048858	cell projection morphogenesis	93	1	0.71	0.00441
23	GO:0120039	plasma membrane bounded cell projection ...	93	1	0.71	0.00441
24	GO:0046034	ATP metabolic process	30	1	0.23	0.00461
25	GO:0006631	fatty acid metabolic process	59	2	0.45	0.00499
26	GO:0016052	carbohydrate catabolic process	21	1	0.16	0.00555
27	GO:0019752	carboxylic acid metabolic process	140	3	1.07	0.00556
28	GO:0043436	oxoacid metabolic process	140	3	1.07	0.00556
29	GO:0032989	cellular component morphogenesis	107	1	0.82	0.00602
30	GO:0006082	organic acid metabolic process	141	3	1.08	0.00651
31	GO:0009653	anatomical structure morphogenesis	253	1	1.93	0.00723
32	GO:0006935	chemotaxis	72	0	0.55	0.00746
33	GO:0098656	anion transmembrane transport	29	0	0.22	0.00776
34	GO:0006928	movement of cell or subcellular componen...	115	0	0.88	0.00822
35	GO:0032502	developmental process	604	9	4.61	0.00895
36	GO:0031175	neuron projection development	99	1	0.76	0.00909
